# The Clinical Medical Librarian's Handbook

**DOI:** 10.5195/jmla.2022.1313

**Published:** 2022-01-01

**Authors:** Paige Scudder

**Affiliations:** 1 paige.n.scudder@dartmouth.edu, Biomedical Libraries, Dartmouth College, Hanover, NH

Aimed with a clear goal of arming library students, practicing librarians, and health care workers with an overview of the medical librarian profession, *The Clinical Medical Librarian's Handbook* is an excellent entry-level read that speaks to the constant evolution of the field. Each chapter is designed to provide background information, applicable case studies or interviews, and suggestions for moving forward.

Starting with a brief history of how the role of a clinical medical librarian came to be and the support systems put in place to create more effective services and resources for medical libraries, the reader learns how the clinician's need for current and reliable information, as well as the patient's need for education, has shaped clinical librarianship. As the chapters progress, this theme remains at the forefront as consumer health, public health, and the resources needed to support each are addressed.

Following a discussion of the inception of the clinical medical librarian, chapter 2 focuses on developing clinical partnerships and their importance in breaking down existing silos:

Silos exist to protect the status quo. Your mission is to introduce new advancements in services to existing customers and build new customer bases, not maintain the status quo. How do you become an effective change agent in environments resistant to transformation? Creating transformational partnerships requires strategic planning (p. 19).

Chapter authors Judy C. Stribling and Antonio P. DeRosa take the time to acknowledge that barriers exist, but it's important to work within and around them. Knowing who you are serving, what their needs are, how their needs are changing, and whether you are able to meet those needs with your existing programming are key pieces of information that Stribling and DeRosa encourage you to identify and address.

Not all clients of a medical library are clinicians, as illustrated in the chapters that follow. *The Clinical Medical Librarian's Handbook* identifies the need for and provides suggestions on forming relationships with clinical learners, residents, nurses, patients, and other key stakeholders. These relationships often take the form of instructor, information provider, and research partner, but ultimately depend on the individual and their needs. To demonstrate this, information is provided about existing programs designed to provide consumer health information to residents and their patients, clinical resources, and general health literacy. When possible, chapter authors provide insight into relevant and specific programs and collaborations as seen in chapter 8, “Journey to Magnet: A Nursing-Librarian Collaboration for Nursing Excellence.”

Even those who have been practicing in the medical librarian field are able to read this book and think about topics in new ways. For example, the author of this review appreciates figure 2.1, as it presents a search strategy worksheet in a way not previously used. Additionally, chapter 9 focuses on educating clinical learners and identifies important considerations to keep in mind when working with novices. This information is both helpful for the individual learning and a refresher to the individual who has been a clinical librarian for a while.

Read this book if you are in library school considering becoming a clinical medical librarian, if you are a practicing librarian seeking to ramp up client support, a health care worker interested in how to best collaborate with your existing library, or an individual hoping to build a medical librarian program. *The Clinical Medical Librarian's Handbook* is a quick and informative read that will teach you about the roles of a medical librarian, how to teach clinical resources, the power of patient-centered care, and where a librarian fits into the team.

